# Targeting cancer cell stiffness and metastasis with clinical therapeutics

**DOI:** 10.1007/s10585-025-10353-2

**Published:** 2025-06-11

**Authors:** Alexa M. Gajda, Raymundo Rodríguez-López, Ekrem Emrah Er

**Affiliations:** Department of Physiology and Biophysics, College of Medicine, University of Illinois at Chicago, Chicago, IL USA

**Keywords:** Cell stiffness, Compliance, Mechanobiology, Mechanotherapeutics, Metastasis, Tumor microenvironment

## Abstract

Tumorigenesis and metastasis of solid tumors are coupled to profound biophysical changes that alter cancer cells’ mechanobiology, critically impacting metastatic progression. In particular, cell stiffness determines the ability of cancer cells to invade surrounding tissues, withstand shear fluid stress and evade immune surveillance. Here, we summarize the biological factors, pathological factors, and therapeutic modalities that affect the mechanobiology of cancer cells. We focus on clinically utilized chemotherapeutics and targeted therapies that show direct and indirect modulation of cancer cells’ stiffness and discuss how these treatments can be used in combination with other treatment modalities to improve patient outcomes. Finally, we list the outstanding challenges in the field and provide a perspective on expanding the clinical utilization of experimental therapeutics that can act as “mechanotherapeutics” by regulating mechanobiology of cancer cells.

## Introduction—correlations between changes in cellular stiffness during the metastatic cascade

Epithelial cell stiffness undergoes profound changes during oncogenic transformation and malignant cancer progression [1]. Here, we adopt the definition of cellular stiffness as the ability of cells to resist compressive stresses [2]. This cellular stiffness is different than bulk tumor tissue stiffness, which is largely influenced by the deposition, composition and the orientation of extracellular matrix (ECM) proteins [3–7]. Bulk tissue stiffness is measured by pre-clinical and clinical methods, such as palpitation, ultrasound elastography and macro-indentation and these methods cannot resolve the contribution of ECM from the contribution of individual cells to tissue stiffness [8–13]. In contrast, measuring stiffness of individual cells requires methods with higher spatial resolution such as atomic force microscopy (AFM), micro-pipette aspiration, and particle tracking microrheology (PTMR). These methodologies have been expertly reviewed elsewhere [14–16]. Each method measures a different parameter that contribute to overall stiffness of the cell and they all have their caveats and advantages [17–28] (Table 1).

**Table 1:** Methods to measure cellular mechanical properties

Technique	Location and resolution	Pros (+)	Cons (−)	References
Magnetic twisting cytometry (MTC)	Usually on the surface of the cell, detects displacements or deformation of 4–5 nm	• Ability to measure the mechanical properties of different cells at the same time • Different probes for specific measurements	• Contact based, and beads may induce global cellular deformation and difficulty in measurements • Information only of the mechanical properties in contact and around the beads	[25, 26]
Particle tracking microrheology (PTM)	Intracellular, ~ 5 nm	• No applied forces necessary for measurements • Micromechancial properties of cells in a physiological environment	• Exogenous beads are inserted into the cells	[28]
Size-normalized acoustic scattering (SNACS)	Measures mechanical properties of whole cell, with deformations < 15 nm	• Fast measurements of single whole cells, so tracking of different stages of the cells is possible • Non-invasive to the cell	• Subcellular measurements are not possible	[22]
Micropipette aspiration (MPA)	On the membrane of the cell, sub-nanometer deformations, spatial resolution based on the camera	• Single and direct cell measurements • Combined with other techniques (e.g. fluorescence, confocal microscopy) to measure different cell mechanical assays (e.g. cell–cell adhesion forces, cell expression mechanical behavior)	• Heterogeneity, anisotropy, and changes of the cells can not be measured • Limited by camera	[17, 20]
Atomic force microscopy (AFM)	On the surface of the cell, ~ 10 nm but can change with tip	• Different tips with different sizes that can be functionalize for different measurements • Different modes for biochemical, topology and mechanical characterization	• Typically measures mechanical properties of the surface of the cell unless indentation depth is increased • Depends on various mathematical models for mechanical characterization	[24]
Optical tweezers (OT)	Intracellular measurements with nanometer scale, depending on beads	• Different modes to measure several mechanical characteristics of the cell (viscoelasticity, different moduli) • Allows for different intracellular mapping	• Requires exogenous beads • Laser and beads could damage cells	[18]
Brillouin microscopy (BM)	Intracellular, > 1 µm	• Label free and non-invasive, safe for cells • Controlled 3D imaging of mechanical properties of the cell is possible	• Based mostly on empirical correlations, biological theoretical interpretation of mechanical signature still on going	[21]
Real-time deformability cytometry (RT-DC)	Measures mechanical properties of whole cell, by analyzing deformations of the size of the cell, in the order of microns	• Fast measurements of single whole cells • Established theory to explain cell deformation • Non-invasive and label free measurements	• Assumptions of a perfectly isotropic sphere in the theory to calculate mechanical properties • Subcellular measurements are not possible	[23, 27]
Electro-mechanical shear flow deformability cytometry (sDC)	Measures mechanical properties of whole cell, by analyzing deformations of the size of the cell, in the order of microns	• To obtain mechanical properties, analysis not only of the deformation of the cell, but also its electrical response to shear forces • Non-invasive, label free measurements	• Subcellular measurements are not possible	[19]

In the classical models of tumor progression, the first steps of cellular transformation are the expression of oncogenes and the loss of tumor suppressor proteins. These proto-oncogenic events collectively lead to an increase in cell stiffness, such that pre-cancerous cells become stiffer than their parental counterparts. Several lines of evidence from PTMR experiments support this notion. For example, in hyperproliferative, but otherwise non-transformed, MCF10A mammary epithelial cells, expression of the oncogene Her2, a member of the Epidermal Growth Factor Receptor (EGFR) family of receptor tyrosine kinases (RTK), and of H-Ras or K-Ras, two oncogenic small GTPases, increases cell stiffness when these cells are grown on pathologically stiffened three-dimensional matrices and under confinement [29–31]. In the same model, knockout of Phosphatase Tensin Homologue (PTEN) further increases cell stiffness [29]. Furthermore, proto-oncogenic Src activation in *Drosophila* epithelium leads to in situ hyperplasia formation and this stage is marked by an elevation in cellular stiffness [32]. These data suggest that the pre-cancerous stage of oncogenic transformation is marked by an increase in cell stiffness.

Conversely, transition from benign tumor cells to invasive cancer, which bestows cancer cells the ability to metastasize and colonize distant organs, is marked by a compliant (i.e. soft) cellular phenotype. This is backed by results from AFM experiments by using human breast cancer biopsies, wherein benign ductal carcinoma in situ (DCIS) samples are marked by elevated stiffness, whereas invasive cancers present with decreased cellular stiffness [33]. ECM content of the intact tumors can influence these AFM measurements, but comparison of several cancer cell lines show that cells with higher metastatic potential are generally softer than non-metastatic counterparts [34–36]. Furthermore, compliant cells separated by microfluidics show enrichment of cancer stem cell gene expression in comparison to stiff cancer cells. Softer cancer cells are also enriched in the expression of epithelial to mesenchymal transition (EMT) genes, which are associated with invasive and metastatic behavior [37, 38]. These softer cancer cells, which are found in the leading edges of collectively migrating cancer cell clusters in 2-dimensional spaces and in 3-dimensional matrices, are thought to be precursor cells for metastatic dissemination [37, 39].

**Fig. 1:**
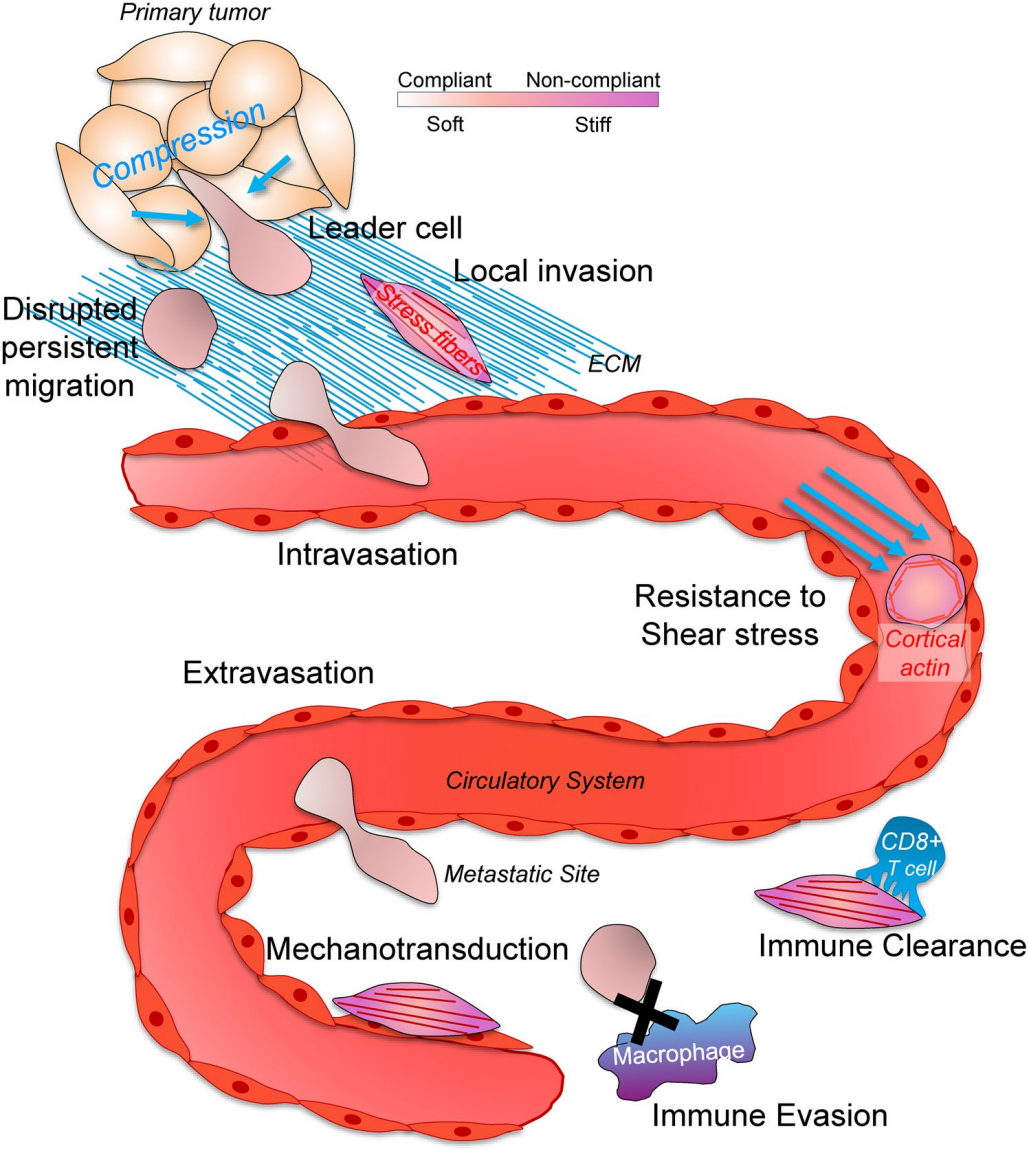
The functional consequences of cell stiffening and softening in metastasis. Cancer cells alter their stiffness in response to changing environmental conditions throughout the metastatic cascade. This mechanoadaptation promotes metastatic dissemination, which means cancer cells have to assume an optimal stiffness value to progress through the metastatic cascade. In contrast, a stiffer phenotype generally exposes cancer cells to immune-mediated clearance by cytotoxic lymphocytes and macrophages

Metastatic dissemination requires cancer cells to navigate the physical confinements of the surrounding ECM and intravasate into and out of the circulation [40, 41]. In this context, compliant cancer cells, especially cells that can perform nuclear deformations (meaning that their nucleus can become softer due to genetic alterations or compressive stress) more readily invade through these constricted spaces for local invasion [42]. This ability is subsequently important for intravasation into and extravasation out of the endothelium for hematogenous dissemination. In general, these studies point to softer phenotypes correlating with higher invasive, migratory and metastatic potential, but it is unlikely that such linear correlations exist in vivo [43, 44]. This is because migratory capacity of cells is likely to be diminished if their softness prevents them from generating traction forces necessary for persistent migration [8]. Furthermore, softer cancer cells cannot withstand destruction by shear stress [45]. Similarly, cells with softer nuclei more readily undergo cell death under shear stress [46]. These studies suggest that the relationship between metastatic phenotypes and cellular stiffness follows a Goldilocks pattern: Cancer cells that can adapt to mechanically challenging environments have the highest metastatic potential (Fig. 1).

While optimal stiffness regulates the metastatic dissemination process, studies to date show a more linear relationship between cancer cell stiffness and their sensitivity to immune-mediated destruction: compliant, softer cells have been shown to be more resistant to targeting by immune cells [47]. For example, increasing the stiffness of melanoma, breast cancer, and lymphoma cells facilitates adoptive T-cell therapy, natural killer (NK) cell-mediated cytotoxicity, and increases the effectiveness of immune checkpoint blockade (ICB) treatments [48–51]. There are several reasons for the improvement of cytotoxic response on stiff target cells: For example, NK cells better orient microtubule organizing centers (MTOCs) for improved secretion of cytotoxic perforin and granzymes when they oppose stiffer surfaces [52]. Cytotoxic T-lymphocytes (CTLs) also show improved force generation and filamentous actin (F-actin) accumulation at the immune synapse when they encounter target cells with elevated membrane tension and cortical stiffness [48]. Furthermore, cytotoxic lymphocytes activate mechanotransduction to a higher magnitude when they encounter stiff target surfaces as judged by the elevated phosphorylation of the zeta-chain-associated protein kinase 70 (ZAP70), by the improved production of pro-apoptotic cytokines, such as tumor necrosis factor (TNF) and interferon gamma (IFNɣ), and by the elevated expression of lymphocyte activation markers such as CD69 and CD25 (encoded by the *IL2RA* gene, interleukin-2 receptor alpha chain) [53, 54]. On the cancer cell side of the immune synapse, elevated membrane tension due to increased cortical stiffness facilitates the physical insertion of perforin pore complexes into the target membranes [55]. Importantly, emerging evidence suggests that disseminated cancer cells that remain dormant in secondary environments assume a softer phenotype to avoid destruction by CTLs and NK cells [56]. Beyond the cytotoxic responses, stiff cancer cells are more readily engulfed by macrophages through phagocytic cup formation, whereas compliant cells are engulfed through the time-consuming trogocytosis process, also known as nibbling [57, 58]. Together, these studies highlight the need for identifying biological and pathological factors that contribute to cellular stiffness for better immune targeting of cancer cells.

## Biological and pathological factors that contribute to cancer cell stiffness

**Fig. 2:**
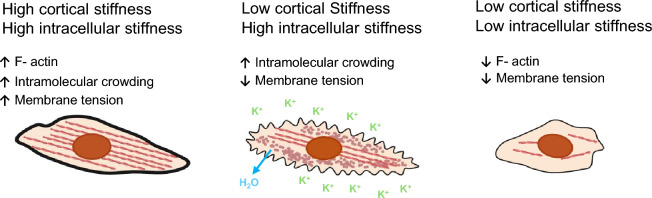
Uncoupling between intracellular stiffness, intramolecular crowding and cortical membrane stiffness under different biological conditions. Intramolecular crowding positively correlates with overall stiffness and membrane stiffness and tension, but under certain circumstances this relationship may be uncoupled. For example, under hypertonic conditions, cells shrivel and lose water, which leads to intramolecular crowding and loss of membrane stiffness and tension. In this scenario, it is not clear which mechanical property of the cancer cell is more dominant in terms of contributing to the metastatic fate. Theoretically increased cytoplasmic stiffness can reduce metastatic potential due to loss of invasive capacity, but decreased membrane stiffness and tension could also promote immune evasion from cytotoxic T-cells and natural killer cells. Figure was created by using BioRender

There are several biological and pathological factors that act in a cell-intrinsic manner to regulate stiffness of cancer cells, such as intramolecular crowding, lipid composition of the plasma membrane and the underlying cytoskeleton [59]. PTMR experiments show that intracellular water influx reduces intramolecular crowding and allows easier diffusion of proteins and thereby reduces cell stiffness [60]. Similarly, hypertonic microenvironments make cancer cells less compliant due to water efflux, elevated intramolecular crowding, and cell shriveling as judged by Brillouin microscopy and electrical deformability cytometry [19, 61, 62]. These data suggest that hypertonic conditions generally promote cellular stiffness, but cell shriveling can also decrease stiffness of the cellular plasma membrane due to loss of membrane tension [63]. For example, incubating endothelial cells with an elevated concentration of extracellular potassium ([K^+^]_e_), which is a hypertonic condition, significantly softens endothelial cells’ membranes as judged by AFM measurements [64, 65]. These data suggest that hypertonic conditions create an uncoupling between intracellular stiffness and the stiffness of the cortical membrane (Fig. 2).

A cell-intrinsic factor that affects cortical cell stiffness without any reported effects on intramolecular crowding is the cholesterol content of the cellular plasma membrane. Tumorigenesis and the subsequent increased cellular metabolism require cholesterol production in cancer cells to sustain their proliferation [66]. Additionally, an optimal level of membrane cholesterol is necessary for the maintenance of cellular stiffness. For example, in endothelial cells, cholesterol extraction from the plasma membrane by the small molecule 2-Hydroxypropyl-β-cyclodextrin increases cell stiffness [67]. Similarly, in hepatocellular carcinoma cells, cholesterol depletion through V-ATPase inhibition by Archazolid A causes cortical stiffening [68]. Furthermore, pharmacologically or genetically depleting plasma membrane cholesterol in melanoma cells elevates their cortical stiffness [48].

A major factor that is involved in both intramolecular crowding and cortical cell stiffness is the cytoskeleton. The cytoskeleton is a collection of fibrillar networks within the cell that determines cellular morphology and architecture in response to various environmental and differentiation cues [59]. The most prominently studied cytoskeletal elements for cell stiffness include microtubules, F-actin, and the actomyosin network. Several small molecule drugs, including Latrunculin A and Cytochalasin D, inhibit F-actin polymerization and disband F-actin stress fibers, significantly reducing the stiffness of cancer cells and fibroblasts. Conversely, promoting F-actin bundling, stress fiber formation, and enhancing actomyosin contractility through treatment with small molecule drugs, such as jasplakinolide and 4-Hydroxyacetophenone, which activates non-muscle myosin IIC (encoded by the *MYH14* gene), increases cell stiffness [39, 49, 69].

**Fig. 3:**
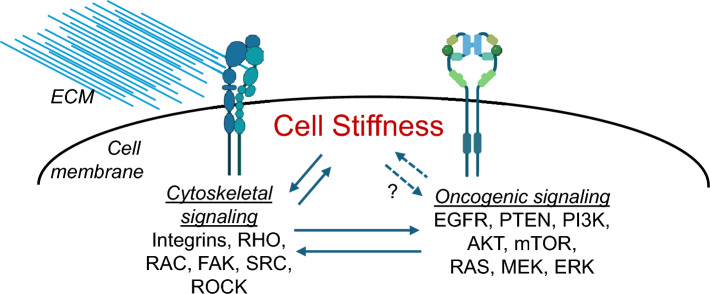
Relationship between cytoskeletal signaling and oncogenic signaling pathways in regulating stiffness of cancer cells. Schematic showing how signaling pathways that are known to directly regulate the cytoskeleton and therefore contribute to cellular stiffness interact with oncogenic signaling pathways exemplified by the EGFR/RAS/MEK/ERK and PI3K/AKT/mTOR pathways. Regulation of cell stiffness by oncogenic signaling pathways requires post-translational modification of the components of the cytoskeletal signaling machinery, but more direct mechanisms may also exist. While cell adhesion and cytoskeletal signaling are known to regulate growth factor signaling pathways commonly altered in cancer, whether cell stiffness biophysically regulates these oncogenic signaling pathways is also unknown. Figure was created by using BioRender

Based on the high level of contribution of F-actin to cellular stiffness, it is conceivable that activation of signaling pathways that promote F-actin bundling and stress fiber formation will also contribute to cell stiffness. Cytoskeletal signaling pathways are controlled by inputs from the integrin family of cell-ECM adhesion molecules and oncogenic signaling pathways [70–73]. For example, increased stiffness of the ECM increases the stiffness of cells through the process of mechanoreciprocity, which involves integrin engagement, activation of focal adhesion kinase (FAK) and downstream Src family of tyrosine kinases, Rho family of GTPases, and Rho associated kinase (ROCK) [74–77]. Perturbing integrin-ECM contact or inhibiting ROCK softens cancer cells [78, 79]. However, FAK knockdown in endothelial cells leads to increased cell stiffening, which highlights the role of FAK in counteracting stress fiber formation by dissembling mature cell adhesions [80]. The oncogenic EGFR, Her2, Ras, PI3K/Akt, and the downstream mTOR pathways all contribute to regulation of cell stiffness either through transcriptional activation of F-actin remodelers or by directly impacting the activity of Src, FAK, and Rho activation in various contexts [70, 72, 73]. Together, these studies highlight how targeting oncogenic signaling pathways can have a significant impact on cytoskeletal signaling and thereby cellular stiffness (Fig. 3).

### Indirect manipulation of cancer cell stiffness using current targeted and chemotherapeutics

The vast majority of current cancer drugs are designed to inhibit cancer cell proliferation, induce apoptosis, or block pathways deemed essential for their survival. Though many of the drugs discussed here have been around for decades, their impact on cancer cell stiffness has only been recently investigated. For example, there are several targeted therapies that inhibit oncogenic signaling pathways involved in proliferation and cell survival [81]. At the apex of oncogenic signaling cascades is EGFR and its inhibition by a monoclonal antibody, Cetuximab, stiffens A549, MDA-MB-231, and MCF-7 lung and breast cancer cells [82–84]. Similarly, the Her2 monoclonal antibody Trastuzamab also stiffens the Her2 + SKBR3 breast cancer cells as judged by micropipette aspiration [85, 86]. Lapatinib, an RTK inhibitor for EGFR, also increases stiffness of MCF-7 and A431 cells in culture [87]. Targeting downstream of EGFR by using Trametinib, a mitogen activated protein kinase kinase (MEK) inhibitor, in MDA-MB-231 breast cancer and MDA-MB-435 melanoma cells causes them to stiffen [88]. Conversely, treatment of A549 cells with another EGFR inhibitor, Gefitinib, softens these cancer cells and reduces the stiffening responses of MCF-7 cells to applied force [89, 90]. These contradictory results suggest that despite targeting the same RTKs and downstream pathways, the mode of action of each therapeutic can produce different effects on cellular stiffness. It is possible that the on-target effect of shutting down MEK and downstream extracellular signal regulated kinase (ERK) signaling could lead to lower F-actin cytoskeleton turn-over and thereby increased stiffening [72]. Alternatively, off-target effects of Gefitinib, such as inhibition of Src or Serine Threonine Kinase 10 (STK10) that are both directly involved in cytoskeletal remodeling, could lead to cellular softening [91, 92].

PI3K/AKT/mTOR pathway inhibitors are in clinical trials and have been approved for treating patients with various malignancies. Application of PI3K/AKT/mTOR inhibitors generally yield a stiffer cancer cell phenotype in vitro. Treatment of the PC-3 prostate cancer cell line, which has activated PI3K/AKT signaling due to homozygous loss of the tumor suppressor PTEN, with the allosteric AKT inhibitor, MK-2206, causes these cells to stiffen [93]. Consistent with the idea that PI3K signaling decreases cellular stiffness, treatment of MCF-7 and MDA-MB-231 cells with Everolimus, an mTOR complex 1 inhibitor, also increases cancer cell stiffness [94]. The observed effect of PI3K/AKT/mTOR inhibitors on cellular stiffness could be explained by the involvement of PI3K signaling in regulating F-actin cytoskeleton dynamics through generation of phosphatidylinositol (3,4,5)-trisphosphate (PIP_3_), which regulates Rho family of GTPases, and through phosphorylation of actin-bundling proteins, but alternative mechanisms that involve regulation of cell cycle and survival cannot be ruled out without experimental testing [70, 95].

FDA-approved agents for the BCR-ABL fusion protein in chronic and acute lymphocytic leukemia include Imatinib, a selective ABL kinase inhibitor, and Dasatinib, a dual ABL and Src kinase inhibitor, making the latter a suitable choice for probing the role of Src in mechanobiology. Treatment of normal podocytes or BRAF mutant A375 melanoma cells with Dasatinib reduces cell stiffness, whereas Imatinib has not been recorded to have such effects [96, 97]. Src activation is coupled to FAK activity during cell adhesion and treatment of MCF7 cells with Defactinib increases cell stiffness, but does not change stiffness of MDA-MB-231 cells [98]. Overall, these studies highlight inhibiting Src and FAK have opposite effects on cellular stiffening and that the broad involvement of these kinases in multiple contexts requires further investigation.

Hormone therapy for hormone positive breast cancers has also been shown to impact cancer cell stiffness. In the MCF-7 model of Estrogen Receptor positive (ER+) breast cancer, treatment of cells with Tamoxifen, a selective estrogen receptor modulator (SERM), causes them to stiffen [85]. Interestingly, evolution of Tamoxifen resistance in the same cell line coincides with cellular softening, suggesting that cellular softening is a marker for Tamoxifen resistance [99]. However, these data do not suggest that cellular softening is a marker of broad drug resistance. Indeed, aromatase inhibitor resistance and resistance to Fulvestrant treatment in the same MCF-7 cell line manifests as epigenetic regulation that ultimately leads to upregulation of Keratin-80, which increases cellular stiffness [100]. Moreover, the use of different aromatase inhibitors targeting the same enzyme does not yield the same stiffening effect. A side-by-side comparison of Letrozole and Exemestane treatment on MCF-7 cells yielded opposite results determined by AFM measurements. Letrozole treatment softened MCF-7 cells while Exemestane stiffened them [94]. These differential results point to the importance of understanding which biomechanical pathways are being impacted downstream of these drugs, regardless of their intended targets.

DNA damaging chemotherapeutics also increase cellular stiffness. Cisplatin treatment of lung, colon, prostate cancer, and melanoma cells increase cellular stiffness [83, 101, 102]. In the case of prostate cancer cells, cisplatin-mediated increase in cell stiffness is coupled to F-actin polymerization [102]. Treatment of leukemic cells with another DNA-damaging agent, daunorubicin, also stiffens cancer cells [103]. DNA damage is known to induce nuclear F-actin polymerization, and this effect could elevate the overall stiffness of the cancer cell, but this idea requires formal testing [104]. Together, these data suggest that the overall growth retardation and tumor suppression in response to drug treatment correlates with an elevated cellular stiffness, but there are also several studies, which demonstrate the opposite phenomenon. For example, the tumor suppressive effect of carboplatin treatment on ovarian cancer organoids correlates with a reduction in their stiffness, suggesting that cell softening is indicative of chemotherapy response [105]. Indeed, in a panel of ovarian cancer cell lines, cisplatin resistance accompanies elevated cancer cell stiffness and making cancer cells more compliant by targeting Rho GTPases restores cisplatin sensitivity [106]. Together, these studies demonstrate that targeted therapies and chemotherapies have an impact on cancer cell stiffness, but whether this is due to direct regulation of F-actin polymerization and cytoskeletal signaling proteins, or due to a broad activation of stress signaling pathways and subsequent cellular toxicity needs further investigation (Table 2).

**Table 2:** Therapeutic treatments that indirectly affect cell stiffness

Drug	Target	Cell type	Effect on stiffness	Method	Reference
Cetuximab	EGFR inhibitor	A594	↑	AFM	[83]
		MDA-MB-231	↑	AFM	[82]
		MCF7	↑	AFM	[82]
Trastuzumab	HER2 inhibitor	SKBR3	↑	micropipette aspiration	[85,86]
Lapatinib	EGFR inhibitor	A431	↑	AFM	[87]
		MCF7	↑	AFM	[87]
Trametinib	MEK inhibitor	MDA-MB-231	↑	AFM	[88]
		MDA-MB-435	↑	AFM	[88]
Gefitinib	EGFR inhibitor	A549	↓	AFM	[89]
		MCF7	↓	traction force microscopy	[90]
MK-2206	AKT inhibitor	PC-3	↑	AFM	[93]
Everolimus	mTOR inhibitor	MCF7	↑	AFM	[94]
		MDA-MB-231	↑	AFM	[94]
Dasatinib	ABL/Src kinase inhibitor	A375	↓	AFM	[96]
Defactinib	FAK inhibitor	MCF7	↑	AFM	[98]
		MDA-MB-231	–	AFM	[98]
Tamoxifen	ER modulator	MCF7	↑	micropipette aspiration	[85]
Letrozole	Aromatase inhibitor	MCF7	↓	AFM	[94]
Exemestane	Aromatase inhibitor	MCF7	↑	AFM	[94]
Cisplatin	Platinum-based chemotherapy	A549	↑	AFM	[83]
		Calu-6	↑	AFM	[83]
		PC-3	↑	AFM	[102]
		B16F10	↑	AFM	[101]
Daunorubicin	DNA-damage chemotherapy	AML	↑	AFM	[103]

### Direct manipulation of cancer cell stiffness using current targeted and chemotherapeutics

In contrast to the previous section, there are several anti-cancer therapies which are designed to directly target cytoskeletal components or motor machinery to either interfere with cell division or impair cell migration (Table 3). This class of drugs is primarily tied to preventing proper polymerization or depolymerization of microtubules or actin filaments, subsequently altering cancer cell stiffness. One of the most commonly used classes of chemotherapeutic drugs for treating patients with solid tumors include taxanes. These drugs, such as paclitaxel and docetaxel, prevent microtubule depolymerization, halting cancer cell mitosis and leading to apoptosis. Though there are some exceptions, this class of drugs generally stiffens cancer cells, which is expected as their on-target effect. For example, docetaxel treatment of prostate cancer cells yielded cells with a stiffer phenotype, as determined by AFM, along with an increased accumulation of tubulin at the periphery of the cells [102]. Similarly, vinca alkaloids, such as vinblastine or vinflunine, block mitosis by binding β tubulin and preventing microtubule polymerization [107]. Surprisingly, vinflunine was found to increase prostate cancer cell stiffness, not through noticeable changes in microtubule networks, but through an increase in actin polymerization around the nucleus of cells, thought to be a resistance mechanism developed by these cells to take advantage of the actin-microtubule internetwork crosstalk [108].

**Table 3:** Therapeutic treatments that directly affect cell stiffness

Drug	Target	Cell type	Effect on stiffness	Method	Reference
Docetaxel	Microtubule depolymerization inhibitor	PNT1A	↑	AFM	[102]
		22Rv1	↑	AFM	[102]
		PC-3	↑	AFM	[102]
Vinflunine	Microtubule polymerization inhibitor	DU145	↑	AFM	[108]
Salinomycin	FAK inhibitor	Liver cancer stem cells	↑	AFM	[116]

Although drugs directly targeting actin polymerization are generally considered highly toxic due to the necessity of this key cytoskeletal component in all cells, healthy and cancerous alike, there are some drugs which specifically target actin bundling and stability [109]. For example, the actin-bundling protein, Fascin-1 is a known promotor of cancer cell migration and its frequent upregulation in various tumor types is well-known to correlate with worse survival outcomes. Interestingly, the use of Fascin-1 inhibitors, such as migrastatins and the repurposed drugs, raltegravir and imipramine, have demonstrated slowed cancer invasion in both in vitro and in vivo models. Though it remains unknown whether these treatments soften tumor cells along with their decreased actin-bundling, there are studies that show Fascin-1 knockdown does decrease glioma cell stiffness [110–113]. Similarly, therapeutic inhibition of the actin-stabilizing and contractility aiding protein, tropomyosin, using TR100, leads to actin disorganization in human melanoma cells [114]. Though genetic knockdown of tropomyosin in neuroblastoma cells yields cells with a softer phenotype determined by AFM, the impact of the inhibitor TR100 on epithelial cancer cell stiffness remains elusive [115]. Unexpectedly, a repurposed antibiotic, salinomycin, has also demonstrated efficacy in slowing liver cancer stem cell invasion by increasing cell stiffness and F-actin formation through inhibition of the FAK-ERK1/2 pathway [116]. Collectively, it is clear that many of the targeted and broadly cytotoxic treatment modalities have effects on cellular stiffness and these effects on cellular stiffness likely contribute to treatment resistance and response.

## Potential contribution of changes in cellular stiffness to clinical therapy response

Emerging data on the regulation of cellular stiffness by clinically relevant therapeutics provides us with a rationale for interrogating the contribution of cellular stiffness to therapy response. For example, the FAK inhibitor, Defactinib, in combination with Pembrolizumab, an ICB antibody, are currently in a clinical trial for treatment of patients with advanced cancers (NCT02546531). This clinical trial was based on the remarkable effect of combining these two treatments in preclinical models [117, 118]. Since FAK inhibition stiffens cancer cells in vitro and increased stiffness improves ICB approaches in pre-clinical studies, it is tempting to speculate that part of the beneficial effect of this combination therapy may be due to alterations in cell stiffness. One could also extrapolate this line of reasoning to interrogate whether AKT inhibition in combination with ICB could produce such beneficial effects in patients. This is especially important because the AKT inhibitor, Capivesartib, was recently approved for use in combination with Fulvestrant in advanced breast cancers [119]. Both of these treatments increase cellular stiffness in vitro, and they could potentially be used in combination with ICB to prevent metastatic relapse by targeting dormant breast cancer cells [93, 100]. The same logic can also apply to the combined use of ICB with nab-paclitaxel, which presumably stiffens cancer cells, in clinical trials for treating metastatic breast cancer [120]. On the downside, tamoxifen has been used as one of the first line treatments for ER+ breast cancer, but tamoxifen resistance emerges with cellular softening. One could argue that ICB treatments would be less effective in tamoxifen resistant breast cancers because the mechanical input into CTL activation would be limited. ROCK inhibitors have also shown a lot of promise in pre-clinical models for treatment of various cancers in combination with ICB, but it could be challenging to use ROCK inhibitors in combination with ICB in patients if ROCK inhibitors’ softening effect on cancer cells negate the mechanical input necessary for CTL activation [121]. However, testing this idea awaits development of better ROCK inhibitors, since the most advanced ROCK inhibitor failed a clinical trial due to poor pharmacokinetic performance [122].

All of the above-mentioned treatments for stiffening cancer cells would also impact the tumor microenvironment. Theoretically, stiffening cancer cells with these therapies could lead to propagation of compressive stresses throughout the tumor microenvironment. These compressive stresses could, inturn, promote pathological ECM deposition by cancer-associated fibroblasts, and thereby limit immune cell infiltration. One avenue for avoiding pathological ECM deposition could be by co-treatment with Renin Angiotensin System inhibitors to suppress fibroblast activation, prevent ECM deposition and allow immune inflitration as in the case of pre-clinical models of glioblastoma and liver metastases by colorectal cancer [123, 124]. The use of Renin Angiotensin System inhibitors highlight a major opportunity for understanding how systemic treatments impact mechanobiology of cancer cells and how we can repurpose some of these FDA-approved treatments in cardiovascular diseases for targeting cancer cell stiffness [125]. For example, there are several K^+^ channel manipulators used in cardiovascular diseases and these can affect cancer cell stiffness [126]. This is particularly important because high levels of extracellular K^+^ are present in tumor interstitial fluid and this high [K^+^]_e_ shuts down cellular K^+^ efflux and softens endothelial cells [127, 128]. If [K^+^]_e_ also softens cancer cells, it would be important to know whether K^+^ channel targeting drugs have similar effects on cancer cells’ stiffness and therefore regulate metastatic and immune-evasive phenotypes. Finally, drugs used for cholesterol management, such as statins, have been in clinical trials for cancer treatment and are likely to have impacts on pre-malignant and dormant metastatic cells through both cancer-intrinsic mechanisms and mechanisms that directly affect T-lymphocyte and cardiovascular biology [129–132].

## Challenges in targeting cellular stiffness

There are major technical challenges in the field of mechanobiology. First, most of the methods used for determining the stiffness of cells, such as PTMR, AFM and Brillouin spectroscopy require a certain level of tissue dissociation, which removes cancer cells from their tumor microenvironment. While there are efforts to determine the stiffness of processed tissue sections, most require cryosectioning and low temperatures are known for dissembling the F-actin cytoskeleton, which is a major contributor to cellular stiffness. One approach to overcome these technical issues is to deploy chemical fixation strategies. In this setting, all biological structures would stiffen, but the relative stiffness differences within the same tissue preparation would remain intact [133].

A second challenge is that it is difficult to pinpoint the precise molecular mechanism that underlies a pharmacological perturbation’s effect on cellular stiffness. This is because biophysical effects are frequently tied to biochemical effects, which will simultaneously change signaling and transcriptional landscapes of the cell. These secondary effects can in turn impact the physical state of the cell by promoting cell cycle entry, metabolic rewiring, senescence and apoptosis. One approach to differentiate between direct biophysical effects versus secondary biochemical effects of these pharmacological agents on cellular stiffness would be to conduct real-time stiffness measurements during the course of the treatment: Direct biophysical effects treatments would be recorded within seconds or minutes, whereas secondary transcriptional effects would emerges hours or days later. Indeed, this is the case for F-actin modifiers and regulators of ion channels that act within seconds to minutes of application.

Beyond the technical challenges, there are also significant conceptual challenges in the field. Accumulating evidence shows that the malignant phenotype is marked by a softer cancer cell phenotype, that when reversed, may lead to inhibition of migration and metastasis. Yet, addressing exceptions to this general notion is a significant challenge in the field. First, not all tumor cells are marked by a decrease in their stiffness in comparison to normal cells. For example, glioblastoma cells have a similar stiffness to non-transformed cells [134]. In fact, increased stiffness of glioblastoma cells is associated with invasive behavior within the brain microenvironment, and this invasive behavior is the reason why debulking treatments, such as surgery and radiation, are never curative in this disease [135]. Furthermore, increasing cellular stiffness in the absence of active immune surveillance can result in undesired effects. For example, cellular stiffening through F-actin polymerization could promote long-term cancer cell dormancy and survival by triggering cell survival pathways typically activated upon cell-ECM adhesion [136, 137]. F-actin polymerization and subsequent cell stiffness can also increase metastasis by promoting resistance to shear stress in blood circulation [45]. Increase in cellular stiffness can also promote metastasis by amplifying traction forces needed for persistent migration [138]. These studies highlight how mechanoadaptation processes pose a significant challenge to use mechanics to better treat cancer [44].

One approach to overcome mechanoadaptation would be to target processes that are fundamentally important in regulating cell stiffness. The problem with this approach is that it could result in adverse effects on multiple tissues. For example, many of the regulators of cancer cell stiffness are likely to be involved in regulating the biophysical properties of the immune system, and biophysical fitness of immune cells is critical for a robust anti-tumor immune response [47]. Thus, identifying and targeting regulators of cellular stiffness that are unique to cancer cells would be an ideal strategy.

Finally, one would have to consider what the ideal clinical stage to target cellular stiffness would be. An attractive approach is deploying these therapeutics in the adjuvant setting after gross debulking of the primary tumor with surgery or during metastatic dormancy. In this setting, mechanoadaptive processes that allow progression of cancer cells through local invasion, survival through shear stresses in circulation and extravasation would be less relevant since the bulk of the primary tumor cells would have been eliminated by surgery. Additionally, disseminated cancer cells would be in a hostile new niche, devoid of an immune suppressive environment and therefore exposed to cytotoxicity by circulating lymphocytes. Indeed, emerging studies show that elevating the stiffness of dormant tumor cells promote their clearance before they can manifest as lethal metastases in pre-clinical studies [56]. In summary, studying fundamental biology behind regulation of cellular stiffness offers novel perspectives into targeting cancer and opens new areas of investigation for determining how commonly used drugs can impact metastatic outcomes in patients.

## Data Availability

No datasets were generated or analysed during the current study.

## References

[1] Er EE, Tello-Lafoz M, Huse M (2022) Mechanoregulation of metastasis beyond the matrix. Cancer Res 82(19):3409–3419 35877197 10.1158/0008-5472.CAN-22-0419 PMC9530650

[2] Radmacher M (2007) Studying the mechanics of cellular processes by atomic force microscopy. Methods Cell Biol 83:347–372 17613316 10.1016/S0091-679X(07)83015-9

[3] Fernandez-Sanchez ME et al (2015) Mechanical induction of the tumorigenic beta-catenin pathway by tumour growth pressure. Nature 523(7558):92–95 25970250 10.1038/nature14329

[4] Stylianopoulos T et al (2012) Causes, consequences, and remedies for growth-induced solid stress in murine and human tumors. Proc Natl Acad Sci U S A 109(38):15101–15108 22932871 10.1073/pnas.1213353109 PMC3458380

[5] Kalli M et al (2018) Solid stress facilitates fibroblasts activation to promote pancreatic cancer cell migration. Ann Biomed Eng 46(5):657–669 29470747 10.1007/s10439-018-1997-7 PMC5951267

[6] Naba A (2024) Mechanisms of assembly and remodelling of the extracellular matrix. Nat Rev Mol Cell Biol 25(11):865–885 39223427 10.1038/s41580-024-00767-3 PMC11931590

[7] Stosser L et al (1985) Dependence of experimental caries in the rat on the sucrose content of the cariogenic diet. Zahn Mund Kieferheilkd Zentralbl 73(5):448–455 2932871

[8] Paszek MJ et al (2005) Tensional homeostasis and the malignant phenotype. Cancer Cell 8(3):241–254 16169468 10.1016/j.ccr.2005.08.010

[9] Samani A, Zubovits J, Plewes D (2007) Elastic moduli of normal and pathological human breast tissues: an inversion-technique-based investigation of 169 samples. Phys Med Biol 52(6):1565–1576 17327649 10.1088/0031-9155/52/6/002

[10] Sigrist RMS et al (2017) Ultrasound elastography: review of techniques and clinical applications. Theranostics 7(5):1303–1329 28435467 10.7150/thno.18650 PMC5399595

[11] Singh MS, Thomas A (2019) Photoacoustic elastography imaging: a review. J Biomed Opt 24(4):1–15 10.1117/1.JBO.24.4.040902 PMC6990059 31041859

[12] Sneider A et al (2022) Deep learning identification of stiffness markers in breast cancer. Biomaterials 285:121540 35537336 10.1016/j.biomaterials.2022.121540 PMC9873266

[13] Xin Y et al (2023) Biophysics in tumor growth and progression: from single mechano-sensitive molecules to mechanomedicine. Oncogene 42(47):3457–3490 37864030 10.1038/s41388-023-02844-x PMC10656290

[14] Hobson CM, Falvo MR, Superfine R (2021) A survey of physical methods for studying nuclear mechanics and mechanobiology. APL Bioeng 5(4):041508 34849443 10.1063/5.0068126 PMC8604565

[15] Wu PH et al (2018) A comparison of methods to assess cell mechanical properties. Nat Methods 15(7):491–498 29915189 10.1038/s41592-018-0015-1 PMC6582221

[16] Wyss HM (2015) Cell mechanics: combining speed with precision. Biophys J 109(10):1997–1998 26588557 10.1016/j.bpj.2015.10.019 PMC4656880

[17] Berardi M et al (2021) Optical interferometry based micropipette aspiration provides real-time sub-nanometer spatial resolution. Commun Biol 4(1):610 34021241 10.1038/s42003-021-02121-1 PMC8140111

[18] Catala-Castro F, Schaffer E, Krieg M (2022) Exploring cell and tissue mechanics with optical tweezers. J Cell Sci 135(15):1 10.1242/jcs.259355 35942913

[19] Chen J et al (2024) Single-cell electro-mechanical shear flow deformability cytometry. Microsyst Nanoeng 10(1):173 39572527 10.1038/s41378-024-00810-5 PMC11582679

[20] Gonzalez-Bermudez B, Guinea GV, Plaza GR (2019) Advances in micropipette aspiration: applications in cell biomechanics, models, and extended studies. Biophys J 116(4):587–594 30683304 10.1016/j.bpj.2019.01.004 PMC6383002

[21] Handler C, Testi C, Scarcelli G (2024) Advantages of integrating Brillouin microscopy in multimodal mechanical mapping of cells and tissues. Curr Opin Cell Biol 88:102341 38471195 10.1016/j.ceb.2024.102341

[22] Kang JH et al (2019) Noninvasive monitoring of single-cell mechanics by acoustic scattering. Nat Methods 16(3):263–269 30742041 10.1038/s41592-019-0326-x PMC6420125

[23] Mietke A et al (2015) Extracting cell stiffness from real-time deformability cytometry: theory and experiment. Biophys J 109(10):2023–2036 26588562 10.1016/j.bpj.2015.09.006 PMC4656812

[24] Muller DJ et al (2021) Atomic force microscopy-based force spectroscopy and multiparametric imaging of biomolecular and cellular systems. Chem Rev 121(19):11701–11725 33166471 10.1021/acs.chemrev.0c00617

[25] Na S et al (2008) Rapid signal transduction in living cells is a unique feature of mechanotransduction. Proc Natl Acad Sci U S A 105(18):6626–6631 18456839 10.1073/pnas.0711704105 PMC2373315

[26] Na S, Wang N (2008) Application of fluorescence resonance energy transfer and magnetic twisting cytometry to quantify mechanochemical signaling activities in a living cell. Sci Signal. 10.1126/scisignal.134pl1 10.1126/scisignal.134pl1 PMC2754283 18728305

[27] Otto O et al (2015) Real-time deformability cytometry: on-the-fly cell mechanical phenotyping. Nat Methods 12(3):199–202 25643151 10.1038/nmeth.3281

[28] Wirtz D (2009) Particle-tracking microrheology of living cells: principles and applications. Annu Rev Biophys 38:301–326 19416071 10.1146/annurev.biophys.050708.133724

[29] Baker EL et al (2010) Cancer cell stiffness: integrated roles of three-dimensional matrix stiffness and transforming potential. Biophys J 99(7):2048–2057 20923638 10.1016/j.bpj.2010.07.051 PMC3042573

[30] Panciera T et al (2020) Reprogramming normal cells into tumour precursors requires ECM stiffness and oncogene-mediated changes of cell mechanical properties. Nat Mater 19(7):797–806 32066931 10.1038/s41563-020-0615-x PMC7316573

[31] Matthews HK et al (2020) Oncogenic signaling alters cell shape and mechanics to facilitate cell division under confinement. Dev Cell 52(5):563-573e3 32032547 10.1016/j.devcel.2020.01.004 PMC7063569

[32] Tavares S et al (2017) Actin stress fiber organization promotes cell stiffening and proliferation of pre-invasive breast cancer cells. Nat Commun 8:15237 28508872 10.1038/ncomms15237 PMC5440822

[33] Plodinec M et al (2012) The nanomechanical signature of breast cancer. Nat Nanotechnol 7(11):757–765 23085644 10.1038/nnano.2012.167

[34] Cross SE et al (2007) Nanomechanical analysis of cells from cancer patients. Nat Nanotechnol 2(12):780–783 18654431 10.1038/nnano.2007.388

[35] Young KM et al (2023) Correlating mechanical and gene expression data on the single cell level to investigate metastatic phenotypes. iScience 26(4):106393 37034996 10.1016/j.isci.2023.106393 PMC10074148

[36] Lv J et al (2021) Cell softness regulates tumorigenicity and stemness of cancer cells. EMBO J 40(2):e106123 33274785 10.15252/embj.2020106123 PMC7809788

[37] Zou H et al (2022) Single cell analysis of mechanical properties and EMT-related gene expression profiles in cancer fingers. iScience 25(3):103917 35252814 10.1016/j.isci.2022.103917 PMC8889141

[38] Yang J et al (2020) Guidelines and definitions for research on epithelial-mesenchymal transition. Nat Rev Mol Cell Biol 21(6):341–352 32300252 10.1038/s41580-020-0237-9 PMC7250738

[39] Han YL et al (2020) Cell swelling, softening and invasion in a three-dimensional breast cancer model. Nat Phys 16(1):101–108 32905405 10.1038/s41567-019-0680-8 PMC7469976

[40] Cox TR, Erler JT (2011) Remodeling and homeostasis of the extracellular matrix: implications for fibrotic diseases and cancer. Dis Model Mech 4(2):165–178 21324931 10.1242/dmm.004077 PMC3046088

[41] Ilina O et al (2020) Cell-cell adhesion and 3D matrix confinement determine jamming transitions in breast cancer invasion. Nat Cell Biol 22(9):1103–1115 32839548 10.1038/s41556-020-0552-6 PMC7502685

[42] Pfeifer CR, Irianto J, Discher DE (2019) Nuclear mechanics and cancer cell migration. Adv Exp Med Biol 1146:117–130 31612457 10.1007/978-3-030-17593-1_8

[43] Nguyen LTS et al (2022) Cancer as a biophysical disease: targeting the mechanical-adaptability program. Biophys J 121(19):3573–3585 35505610 10.1016/j.bpj.2022.04.039 PMC9617128

[44] Gensbittel V et al (2021) Mechanical adaptability of tumor cells in metastasis. Dev Cell 56(2):164–179 33238151 10.1016/j.devcel.2020.10.011

[45] Moose DL et al (2020) Cancer cells resist mechanical destruction in circulation via RhoA/actomyosin-dependent mechano-adaptation. Cell Rep 30(11):3864-3874e6 32187555 10.1016/j.celrep.2020.02.080 PMC7219793

[46] Mitchell MJ et al (2015) Lamin A/C deficiency reduces circulating tumor cell resistance to fluid shear stress. Am J Physiol Cell Physiol 309(11):C736–C746 26447202 10.1152/ajpcell.00050.2015 PMC4725441

[47] Mittelheisser V et al (2024) Evidence and therapeutic implications of biomechanically regulated immunosurveillance in cancer and other diseases. Nat Nanotechnol 19(3):281–297 38286876 10.1038/s41565-023-01535-8

[48] Lei K et al (2021) Cancer-cell stiffening via cholesterol depletion enhances adoptive T-cell immunotherapy. Nat Biomed Eng 5(12):1411–1425 34873307 10.1038/s41551-021-00826-6 PMC7612108

[49] Liu Y et al (2021) Cell softness prevents cytolytic T-cell killing of tumor-repopulating cells. Cancer Res 81(2):476–488 33168645 10.1158/0008-5472.CAN-20-2569

[50] Tello-Lafoz M et al (2021) Cytotoxic lymphocytes target characteristic biophysical vulnerabilities in cancer. Immunity 54(5):1037-1054e7 33756102 10.1016/j.immuni.2021.02.020 PMC8119359

[51] Zhou Y et al (2024) Cell softness renders cytotoxic T lymphocytes and T leukemic cells resistant to perforin-mediated killing. Nat Commun 15(1):1405 38360940 10.1038/s41467-024-45750-w PMC10869718

[52] Friedman D et al (2021) Natural killer cell immune synapse formation and cytotoxicity are controlled by tension of the target interface. J Cell Sci. 10.1242/jcs.258570 10.1242/jcs.258570 PMC8077183 33712452

[53] Blumenthal D et al (2020) Mouse T cell priming is enhanced by maturation-dependent stiffening of the dendritic cell cortex. Elife. 10.7554/eLife.55995 10.7554/eLife.55995 PMC7417170 32720892

[54] Judokusumo E et al (2012) Mechanosensing in T lymphocyte activation. Biophys J 102(2):L5-7 22339876 10.1016/j.bpj.2011.12.011 PMC3260692

[55] Basu R et al (2016) Cytotoxic T cells use mechanical force to potentiate target cell killing. Cell 165(1):100–110 26924577 10.1016/j.cell.2016.01.021 PMC4808403

[56] Wang Z et al (2024) TGF-beta induces an atypical EMT to evade immune mechanosurveillance in lung adenocarcinoma dormant metastasis. bioRxiv 51:4712 10.1038/s43018-025-01094-y PMC12858408 41492090

[57] Settle AH et al (2024) beta2 integrins impose a mechanical checkpoint on macrophage phagocytosis. Nat Commun 15(1):8182 39294148 10.1038/s41467-024-52453-9 PMC11411054

[58] Cornell CE et al (2024) Target cell tension regulates macrophage trogocytosis. BioRxiv 88:1 10.1038/s41556-025-01807-6 PMC12716991 41387582

[59] Galie PA, Georges PC, Janmey PA (2022) How do cells stiffen? Biochem J 479(17):1825–1842 36094371 10.1042/BCJ20210806

[60] Guo M et al (2017) Cell volume change through water efflux impacts cell stiffness and stem cell fate. Proc Natl Acad Sci U S A 114(41):E8618–E8627 28973866 10.1073/pnas.1705179114 PMC5642688

[61] Zhang J et al (2023) Rapid biomechanical imaging at low irradiation level via dual line-scanning Brillouin microscopy. Nat Methods 20(5):677–681 36894684 10.1038/s41592-023-01816-z PMC10363327

[62] Scarcelli G et al (2015) Noncontact three-dimensional mapping of intracellular hydromechanical properties by Brillouin microscopy. Nat Methods 12(12):1132–1134 26436482 10.1038/nmeth.3616 PMC4666809

[63] Pietuch A, Bruckner BR, Janshoff A (2013) Membrane tension homeostasis of epithelial cells through surface area regulation in response to osmotic stress. Biochim Biophys Acta 1833(3):712–722 23178740 10.1016/j.bbamcr.2012.11.006

[64] Callies C et al (2011) Membrane potential depolarization decreases the stiffness of vascular endothelial cells. J Cell Sci 124(Pt 11):1936–1942 21558418 10.1242/jcs.084657

[65] Oberleithner H et al (2009) Potassium softens vascular endothelium and increases nitric oxide release. Proc Natl Acad Sci U S A 106(8):2829–2834 19202069 10.1073/pnas.0813069106 PMC2637279

[66] Xiao M et al (2023) Functional significance of cholesterol metabolism in cancer: from threat to treatment. Exp Mol Med 55(9):1982–1995 37653037 10.1038/s12276-023-01079-w PMC10545798

[67] Byfield FJ et al (2004) Cholesterol depletion increases membrane stiffness of aortic endothelial cells. Biophys J 87(5):3336–3343 15347591 10.1529/biophysj.104.040634 PMC1304801

[68] Bartel K et al (2017) V-ATPase inhibition increases cancer cell stiffness and blocks membrane related Ras signalling—a new option for HCC therapy. Oncotarget 8(6):9476–9487 28036299 10.18632/oncotarget.14339 PMC5354746

[69] Bryan DS et al (2020) 4-Hydroxyacetophenone modulates the actomyosin cytoskeleton to reduce metastasis. Proc Natl Acad Sci U S A 117(36):22423–22429 32848073 10.1073/pnas.2014639117 PMC7486788

[70] Deng S et al (2022) PI3K/AKT signaling tips the balance of cytoskeletal forces for cancer progression. Cancers 14(7):1562 35406424 10.3390/cancers14071652 PMC8997157

[71] Kechagia JZ, Ivaska J, Roca-Cusachs P (2019) Integrins as biomechanical sensors of the microenvironment. Nat Rev Mol Cell Biol 20(8):457–473 31182865 10.1038/s41580-019-0134-2

[72] Samson SC, Khan AM, Mendoza MC (2022) ERK signaling for cell migration and invasion. Front Mol Biosci 9:998475 36262472 10.3389/fmolb.2022.998475 PMC9573968

[73] Soriano O et al (2021) The crossroads between RAS and RHO signaling pathways in cellular transformation, motility and contraction. Genes 12(6):819 34071831 10.3390/genes12060819 PMC8229961

[74] Sawada Y et al (2006) Force sensing by mechanical extension of the Src family kinase substrate p130Cas. Cell 127(5):1015–1026 17129785 10.1016/j.cell.2006.09.044 PMC2746973

[75] van Helvert S, Storm C, Friedl P (2018) Mechanoreciprocity in cell migration. Nat Cell Biol 20(1):8–20 29269951 10.1038/s41556-017-0012-0 PMC5943039

[76] Wozniak MA et al (2003) ROCK-generated contractility regulates breast epithelial cell differentiation in response to the physical properties of a three-dimensional collagen matrix. J Cell Biol 163(3):583–595 14610060 10.1083/jcb.200305010 PMC2173660

[77] Cabodi S et al (2010) Integrin signalling adaptors: not only figurants in the cancer story. Nat Rev Cancer 10(12):858–870 21102636 10.1038/nrc2967

[78] Srinivasan S et al (2017) Blockade of Rho-associated protein kinase (ROCK) inhibits the contractility and invasion potential of cancer stem like cells. Oncotarget 8(13):21418–21428 28199964 10.18632/oncotarget.15248 PMC5400594

[79] Li X et al (2021) Nanoscale surface topography reduces focal adhesions and cell stiffness by enhancing integrin endocytosis. Nano Lett 21(19):8518–8526 34346220 10.1021/acs.nanolett.1c01934 PMC8516714

[80] Webb DJ et al (2004) FAK-Src signalling through paxillin, ERK and MLCK regulates adhesion disassembly. Nat Cell Biol 6(2):154–161 14743221 10.1038/ncb1094

[81] Waarts MR et al (2022) Targeting mutations in cancer. J Clin Invest. 10.1172/JCI154943 10.1172/JCI154943 PMC9012285 35426374

[82] Azadi S et al (2019) Modulating cancer cell mechanics and actin cytoskeleton structure by chemical and mechanical stimulations. J Biomed Mater Res A 107(8):1569–1581 30884131 10.1002/jbm.a.36670

[83] Rezaei I, Sadeghi A (2023) The effects of cetuximab and cisplatin anti-cancer drugs on the mechanical properties of the lung cancerous cells using atomic force microscope. Biochem Cell Biol 101(6):531–537 37437307 10.1139/bcb-2022-0322

[84] Zhang Q et al (2018) Evaluating the efficacy of the anticancer drug cetuximab by atomic force microscopy. RSC Adv 8(39):21793–21797 35541738 10.1039/c8ra03215g PMC9081852

[85] Metsiou DN et al (2022) Adhesion strength and anti-tumor agents regulate vinculin of breast cancer cells. Front Oncol 12:811508 36052248 10.3389/fonc.2022.811508 PMC9424896

[86] Metsiou DN et al (2019) The impact of anti-tumor agents on ER-positive MCF-7 and HER2-positive SKBR-3 breast cancer cells biomechanics. Ann Biomed Eng 47(8):1711–1724 31098800 10.1007/s10439-019-02284-3

[87] Azadi S et al (2016) Epidermal growth factor receptor targeting alters gene expression and restores the adhesion function of cancerous cells as measured by single cell force spectroscopy. Mol Cell Biochem 423(1–2):129–139 27696309 10.1007/s11010-016-2831-x

[88] Rudzka DA et al (2019) Migration through physical constraints is enabled by MAPK-induced cell softening via actin cytoskeleton re-organization. J Cell Sci. 10.1242/jcs.224071 10.1242/jcs.224071 PMC6589089 31152052

[89] Bernardes N et al (2016) Modulation of membrane properties of lung cancer cells by azurin enhances the sensitivity to EGFR-targeted therapy and decreased beta1 integrin-mediated adhesion. Cell Cycle 15(11):1415–1424 27096894 10.1080/15384101.2016.1172147 PMC4934055

[90] Muhamed I et al (2016) E-cadherin-mediated force transduction signals regulate global cell mechanics. J Cell Sci 129(9):1843–1854 26966187 10.1242/jcs.185447 PMC4893802

[91] Brauer NR et al (2024) Non-kinase off-target inhibitory activities of clinically-relevant kinase inhibitors. Eur J Med Chem 275:116540 38852338 10.1016/j.ejmech.2024.116540 PMC11243610

[92] Verma N et al (2016) Identification of gefitinib off-targets using a structure-based systems biology approach; their validation with reverse docking and retrospective data mining. Sci Rep 6:33949 27653775 10.1038/srep33949 PMC5032012

[93] Ren J et al (2015) An atomic force microscope study revealed two mechanisms in the effect of anticancer drugs on rate-dependent young’s modulus of human prostate cancer cells. PLoS ONE 10(5):e0126107 25932632 10.1371/journal.pone.0126107 PMC4416805

[94] Mohammadi E et al (2021) Chemical inhibitor anticancer drugs regulate mechanical properties and cytoskeletal structure of non-invasive and invasive breast cancer cell lines: study of effects of letrozole, exemestane, and everolimus. Biochem Biophys Res Commun 565:14–20 34087508 10.1016/j.bbrc.2021.05.083

[95] Campa CC et al (2015) Crossroads of PI3K and Rac pathways. Small GTPases 6(2):71–80 25942647 10.4161/21541248.2014.989789 PMC4601376

[96] Calizo RC et al (2019) Disruption of podocyte cytoskeletal biomechanics by dasatinib leads to nephrotoxicity. Nat Commun 10(1):2061 31053734 10.1038/s41467-019-09936-x PMC6499885

[97] Logue JS, Cartagena-Rivera AX, Chadwick RS (2018) c-Src activity is differentially required by cancer cell motility modes. Oncogene 37(16):2104–2121 29379163 10.1038/s41388-017-0071-5 PMC5906457

[98] Choi J, Park S (2022) A nanomechanical strategy involving focal adhesion kinase for overcoming drug resistance in breast cancer. Nanomedicine 43:102559 35390528 10.1016/j.nano.2022.102559

[99] Zbiral B et al (2023) Characterization of breast cancer aggressiveness by cell mechanics. Int J Mol Sci 24(15):12208 37569585 10.3390/ijms241512208 PMC10418463

[100] Perone Y et al (2019) SREBP1 drives Keratin-80-dependent cytoskeletal changes and invasive behavior in endocrine-resistant ERalpha breast cancer. Nat Commun 10(1):2115 31073170 10.1038/s41467-019-09676-y PMC6509342

[101] Kung ML et al (2016) Nanoscale characterization illustrates the cisplatin-mediated biomechanical changes of B16–F10 melanoma cells. Phys Chem Chem Phys 18(10):7124–7131 26886764 10.1039/c5cp07971c

[102] Raudenska M et al (2019) Cisplatin enhances cell stiffness and decreases invasiveness rate in prostate cancer cells by actin accumulation. Sci Rep 9(1):1660 30733487 10.1038/s41598-018-38199-7 PMC6367361

[103] Lam WA, Rosenbluth MJ, Fletcher DA (2007) Chemotherapy exposure increases leukemia cell stiffness. Blood 109(8):3505–3508 17179225 10.1182/blood-2006-08-043570 PMC1852256

[104] Belin BJ, Lee T, Mullins RD (2015) DNA damage induces nuclear actin filament assembly by Formin -2 and Spire-(1/2) that promotes efficient DNA repair. Elife 4:e07735 26287480 10.7554/eLife.07735 PMC4577826

[105] Conrad C et al (2019) Mechanical characterization of 3D ovarian cancer nodules using brillouin confocal microscopy. Cell Mol Bioeng 12(3):215–226 31719911 10.1007/s12195-019-00570-7 PMC6816613

[106] Sharma S et al (2014) The role of Rho GTPase in cell stiffness and cisplatin resistance in ovarian cancer cells. Integr Biol (Camb) 6(6):611–617 24718685 10.1039/c3ib40246k

[107] Cermak V et al (2020) Microtubule-targeting agents and their impact on cancer treatment. Eur J Cell Biol 99(4):151075 32414588 10.1016/j.ejcb.2020.151075

[108] Kubiak A et al (2021) Stiffening of DU145 prostate cancer cells driven by actin filaments—microtubule crosstalk conferring resistance to microtubule-targeting drugs. Nanoscale 13(12):6212–6226 33885607 10.1039/d0nr06464e

[109] Ong MS et al (2020) Cytoskeletal proteins in cancer and intracellular stress: a therapeutic perspective. Cancers 12(1):238 31963677 10.3390/cancers12010238 PMC7017214

[110] Alburquerque-Gonzalez B et al (2021) The FDA-approved antiviral raltegravir inhibits fascin1-dependent invasion of colorectal tumor cells in vitro and in vivo. Cancers 13(4):861 33670655 10.3390/cancers13040861 PMC7921938

[111] Alburquerque-Gonzalez B et al (2020) New role of the antidepressant imipramine as a Fascin1 inhibitor in colorectal cancer cells. Exp Mol Med 52(2):281–292 32080340 10.1038/s12276-020-0389-x PMC7062870

[112] Chen L et al (2010) Migrastatin analogues target fascin to block tumour metastasis. Nature 464(7291):1062–1066 20393565 10.1038/nature08978 PMC2857318

[113] Sarantelli E et al (2023) Fascin-1 in cancer cell metastasis: old target-new insights. Int J Mol Sci. 10.3390/ijms241411253 10.3390/ijms241411253 PMC10379093 37511011

[114] Stehn JR et al (2013) A novel class of anticancer compounds targets the actin cytoskeleton in tumor cells. Cancer Res 73(16):5169–5182 23946473 10.1158/0008-5472.CAN-12-4501

[115] Jalilian I et al (2015) Cell elasticity is regulated by the tropomyosin isoform composition of the actin cytoskeleton. PLoS ONE 10(5):e0126214 25978408 10.1371/journal.pone.0126214 PMC4433179

[116] Sun J et al (2017) Salinomycin attenuates liver cancer stem cell motility by enhancing cell stiffness and increasing F-actin formation via the FAK-ERK1/2 signalling pathway. Toxicology 384:1–10 28395993 10.1016/j.tox.2017.04.006

[117] Coppola S et al (2017) A mechanopharmacology approach to overcome chemoresistance in pancreatic cancer. Drug Resist Updat 31:43–51 28867243 10.1016/j.drup.2017.07.001

[118] Jiang H et al (2016) Targeting focal adhesion kinase renders pancreatic cancers responsive to checkpoint immunotherapy. Nat Med 22(8):851–860 27376576 10.1038/nm.4123 PMC4935930

[119] Nierengarten MB (2024) FDA approves capivasertib with fulvestrant for breast cancer. Cancer 130(6):835–836 38396318 10.1002/cncr.35238

[120] Schmid P et al (2018) Atezolizumab and nab-paclitaxel in advanced triple-negative breast cancer. N Engl J Med 379(22):2108–2121 30345906 10.1056/NEJMoa1809615

[121] Barcelo J, Samain R, Sanz-Moreno V (2023) Preclinical to clinical utility of ROCK inhibitors in cancer. Trends Cancer 9(3):250–263 36599733 10.1016/j.trecan.2022.12.001

[122] McLeod R et al (2020) First-in-human study of AT13148, a dual ROCK-AKT inhibitor in patients with solid tumors. Clin Cancer Res 26(18):4777–4784 32616501 10.1158/1078-0432.CCR-20-0700 PMC7611345

[123] Datta M et al (2023) Losartan controls immune checkpoint blocker-induced edema and improves survival in glioblastoma mouse models. Proc Natl Acad Sci U S A 120(6):e2219199120 36724255 10.1073/pnas.2219199120 PMC9963691

[124] Shen Y et al (2020) Reduction of liver metastasis stiffness improves response to bevacizumab in metastatic colorectal cancer. Cancer Cell 37(6):800-817e7 32516590 10.1016/j.ccell.2020.05.005

[125] Unger T (2002) The role of the renin-angiotensin system in the development of cardiovascular disease. Am J Cardiol 89(2A):3A-9A 10.1016/s0002-9149(01)02321-9 11835903

[126] Sobey CG (2001) Potassium channel function in vascular disease. Arterioscler Thromb Vasc Biol 21(1):28–38 11145930 10.1161/01.atv.21.1.28

[127] Eil R et al (2016) Ionic immune suppression within the tumour microenvironment limits T cell effector function. Nature 537(7621):539–543 27626381 10.1038/nature19364 PMC5204372

[128] Tan JWY et al (2020) In vivo photoacoustic potassium imaging of the tumor microenvironment. Biomed Opt Express 11(7):3507–3522 33014547 10.1364/BOE.393370 PMC7510904

[129] Yan C et al (2023) Exhaustion-associated cholesterol deficiency dampens the cytotoxic arm of antitumor immunity. Cancer Cell 41(7):1276-1293e11 37244259 10.1016/j.ccell.2023.04.016

[130] Jiang W et al (2021) Statins: a repurposed drug to fight cancer. J Exp Clin Cancer Res 40(1):241 34303383 10.1186/s13046-021-02041-2 PMC8306262

[131] Jouve JL et al (2019) Pravastatin combination with sorafenib does not improve survival in advanced hepatocellular carcinoma. J Hepatol 71(3):516–522 31125576 10.1016/j.jhep.2019.04.021

[132] Kim ST et al (2014) Simvastatin plus capecitabine-cisplatin versus placebo plus capecitabine-cisplatin in patients with previously untreated advanced gastric cancer: a double-blind randomised phase 3 study. Eur J Cancer 50(16):2822–2830 25218337 10.1016/j.ejca.2014.08.005

[133] Calo A et al (2020) Spatial mapping of the collagen distribution in human and mouse tissues by force volume atomic force microscopy. Sci Rep 10(1):15664 32973235 10.1038/s41598-020-72564-9 PMC7518416

[134] Onwudiwe K et al (2024) Single-cell mechanical assay unveils viscoelastic similarities in normal and neoplastic brain cells. Biophys J 123(9):1098–1105 38544410 10.1016/j.bpj.2024.03.034 PMC11079864

[135] Monzo P et al (2021) Adaptive mechanoproperties mediated by the formin FMN1 characterize glioblastoma fitness for invasion. Dev Cell 56(20):2841-2855e8 34559979 10.1016/j.devcel.2021.09.007

[136] Zhao B et al (2012) Cell detachment activates the Hippo pathway via cytoskeleton reorganization to induce anoikis. Genes Dev 26(1):54–68 22215811 10.1101/gad.173435.111 PMC3258966

[137] Olson EN, Nordheim A (2010) Linking actin dynamics and gene transcription to drive cellular motile functions. Nat Rev Mol Cell Biol 11(5):353–365 20414257 10.1038/nrm2890 PMC3073350

[138] Kim TH et al (2016) Cancer cells become less deformable and more invasive with activation of beta-adrenergic signaling. J Cell Sci 129(24):4563–4575 27875276 10.1242/jcs.194803 PMC5201020

